# Legget-Garg inequality for a quantum model of ion channels

**DOI:** 10.1038/s41598-026-57853-z

**Published:** 2026-06-18

**Authors:** Paulina Trybek, Łukasz Machura, Jerzy Dajka

**Affiliations:** 1 Institute of Physics, University of Silesia in Katowice, 40-007 Katowice, Poland; 2 The Professor Tadeusz Widła Interdisciplinary Research Centre for Forensic Science and Legislation, University of Silesia in Katowice, 40-007 Katowice, Poland

**Keywords:** Optics and photonics, Physics

## Abstract

Temporal quantum correlations, revealed by violations of the Leggett–Garg inequalities (LGIs), offer a powerful framework to probe nonclassical dynamics in conceptual models, including those inspired by the living systems. Here, we examine a three-state representation of ionic transport through the selectivity filter, previously presented by Seifi et al. Using this model, we systematically investigate how different scenarios, such as initial state preparation, the specification of measurement basis, hopping dynamics, and environmental fluctuations, affect the emergence of temporal nonclassicality. The choice of measurement basis is found to play a decisive role in shaping temporal quantum correlations. Our analysis reveals that the strongest LGI violations occur for intermediate model configurations, which represent a short-lived state where a potassium ion is poised for release while still coupled to surrounding water sites, whereas entry and exit configurations exhibit weaker quantum signatures. Static fluctuations of the hopping parameters confine LGI violations to shorter time windows, while dynamic decoherence suppresses them irrespective of the hopping amplitude. Overall, our findings demonstrate that LGI-based analyses can identify stages where temporal quantum correlations are most significant, highlighting the intermediate knock-on configuration as a promising target for future studies of intrinsically quantum effects in ion-conduction phenomena.

## Introduction

Biological systems are in essence physical, and since quantum mechanics underlies many physical processes, it is natural to investigate whether quantum effects might also shape our understanding of complex biosystems. However, theoretical reductionism, well established in physics and chemistry, remains controversial in many areas of biology^1^. Nevertheless, numerous attempts to interpret biological phenomena in quantum-mechanical terms have been pursued, ranging from molecular mechanisms in genomics to more speculative questions such as consciousness^2^, thereby laying the foundation for quantum biology^3^ as a well-established subdiscipline of the natural sciences. Over the years, this field has been consolidated through compelling theoretical arguments and experimental indications of nonclassical properties, including superposition, coherence, entanglement, and tunneling. Examples in which quantum effects have been invoked include energy transfer in photosynthesis, enzymatic catalysis, olfaction, and even higher-order cognitive processes^4–7^. Exploring biological mechanisms that operate at the intersection of quantum and classical physics can thus deepen our understanding of these processes and provide more refined physical descriptions.

Within this broader framework, fundamental processes, such as charge transfer in ion channels, can serve as illustrative examples of how quantum effects might influence biological functions at an elementary level. The mechanisms of ion transmission across the cell membrane have been extensively investigated over the decades, with physics playing a particularly crucial role and driving significant progress. In studying the permeation properties of ion channels, Poisson-Nernst-Planck theory, Brownian Dynamics, or Molecular Dynamics simulations have been widely considered as principal tools for modeling the properties of ionic transport phenomena. Numerous articles provide an overview of the role of physical theories and models in elucidating key ion channel mechanisms, including permeation, selectivity, and channel gating^8–10^. The advancement of numerical methods, coupled with the growth of computational resources, led to the continuous development of novel theoretical models that incorporate additional factors, such as the geometric structure of the channel^11^, ion-ion or ion-environment interactions^12^, or the signification of the hydrophobic gating mechanism in ionic transfer^13^. It is unfeasible to reference even a fraction of the research that has addressed the new theories or computational methods to specific types of biological or artificial ion channels. Despite years of extensive interdisciplinary studies, the mechanism of ion permeation still remains a central focus of ongoing theoretical and experimental investigations. Current efforts aim to clarify the phenomenon, with particular attention to the critical ion differentiation taking place within the selectivity filter–a structure only a few angstroms wide. The ion channel selectivity refers to the ability to conduct a particular type of ion. The explanation for the unique nature of this phenomenon takes into account thermodynamic selectivity analysis^14^, prevailing theories on the structure of the ion channel pore, or the statistical theory of selectivity and conductivity^15–17^. Several studies propose potential research directions, underscoring the importance of combining various branches of physics, including statistical and quantum mechanics, as potential areas where significant advances in the physical mechanisms governing ion channels are anticipated^18,19^. The idea of applying quantum theory to ion channels has already been discussed in numerous publications. Kariev et al. indicated that critical processes in ion channels, including charge transfer, should be integrated with quantum modeling^20^. Several studies applying quantum theory to ion channels aim to clarify the mechanisms underlying ion selectivity. For example, Salari et al.^21^ suggest that quantum interference may help explain this process, while Vaziri et al.^22^ propose that the selectivity filter could exhibit quantum coherence, potentially influencing both ion conduction and specificity.

In a recent study, Seifi et al.^23^ explored the role of quantum coherence in processes that occur within the selective filter. They specifically examined how coherence is related with the *hopping rate*, defined as the transition rate at which ions overcome energy barriers between different configurations during the ionic conduction process. Building on this work, the same authors introduced a model addressing decoherence and loss of quantum effects in ion channels^24^. Similarly, another recent study suggests that increased coherence can significantly enhance ion channel conductance^25^. The growing application of quantum theories to ion transport highlights the limitations of classical models in fully capturing the mechanisms underlying channel conductance and selectivity. Although purely structural considerations often suffice to explain the key characteristics of some selected ion conduction problems, the highly flexible nature of ion channels indicates that subtle effects can substantially influence their behavior^26^. Because ion permeation dynamics represents a molecular-scale transport problem, non-trivial contributions from quantum mechanical features are plausible^27^. Despite skepticism, transient quantum effects, such as superposition, coherence, or entanglement, may play a functional role in biological systems. In parallel with these quantum-inspired approaches, it remains crucial to examine the models themselves carefully to gain a deeper insight into the underlying processes responsible for their quantum nature.

An example of a quantum-mechanical description of an ion channel was recently proposed in the aforementioned study^23^, where the authors introduced a seemingly simple three-state model of the selective filter mechanism. Despite its phenomenological character, this model provides a framework for interpreting nontrivial aspects of ion transport in quantum-mechanical terms. Building on this approach, the present work investigates a corresponding scheme, with a focus on Leggett-Garg inequalities (LGIs)^28^, which provide a criterion for detecting nonclassical character of temporal correlations, with the aim of determining the extent to which quantumness manifests in the ion dynamics described by the model. In our approach, we go a step further to test whether the quantum representation of the ion channel shows temporal correlations that violate a Leggett–Garg inequality, providing insight into possible quantum features of selective ion transport.

Quantum correlations, both spatial and temporal, are among the features that lie at the core of the non-triviality of the quantum world. Quantum entanglement^29^ (and other types of spatial correlations^30^) are useful resources for quantum information. One of the most powerful methods of entanglement detection^31^ is related to the violation of various forms of Bell inequalities^32^, indicating quantumness of spatially extended multi-partite systems. However, recent studies on quantum correlations *in time* show that they are not less interesting than those that occur in space^33^. In particular, non-classical time correlations between outcomes of measurements realized at different time instants are present in the *violation of the Leggett–Garg inequalities*. Although the Leggett–Garg and Bell scenarios can formally be unified^34^, LGIs and their violations can quantify quantum transport as a particular form of time evolution of quantum systems. In Ref^33^., an up-to-date review of both basic theoretical and experimental achievements related to LGIs is provided. Contrary to the classical world, quantum mechanics makes violation of various LGIs possible. Experimental testing of LGIs and the search for their potential violation are subtle, due to the requirement of non-invasive measurements of the relevant quantity and, in some cases, the “macroscopicity” of quantum states^33^. The LGIs are also intrinsically interesting in microscopic systems^35,36^, so the second requirement is often relaxed. Certain conceptual challenges, such as the “clumsiness loophole,” still remain^33^. To avoid confusion, we simply assert that the violation of an LGI serves as a hallmark of the breakdown of Leggett–Garg realism in the system under consideration. In this way, we aim to capture the fundamental issues associated with the proper interpretation of LGI violations cf. Ref^37^.. For clarity, we emphasize that a violation of the LGI, as defined in standard formulations^33^, provides a clear signature of the departure from classical, realistic behavior in the considered system, encapsulating the conceptual challenges inherent in interpreting such violations cf. Ref^37^.

The proposed strategy is situated within the broader context of temporal quantum correlations and their potential relevance for biological systems. We systematically examine the robustness of these correlations under variations in measurement bases, initial states, and stochastic modifications of the hopping amplitudes, which determine the energy barriers between different model configurations. Environmental influences are incorporated through a controllable dissipation rate in the Lindblad master equation, allowing a detailed assessment of how open-system dynamics affects nonclassical temporal correlations. This framework enables the analysis of key scenarios that may impact the preservation of quantum properties in the modeled system. Remarkably, very few studies have addressed violations of Leggett–Garg inequalities in the context of biologically inspired quantum systems. A notable example is the work on the Fenna–Matthews–Olson complex^38^, which investigates the coherent transfer of excitations from chlorosome antennae to the reaction center and demonstrates that some temporal correlations can persist even under significant decoherence. Thus, our approach provides a significant foundation for the potential implementation of LGi in biologically motivated contexts.

We organize this paper as follows. Section *Methods*starts with a concise overview of the model proposed by Seifi et al^23^., followed by an introduction to the formalism of Leggett–Garg inequalities. Next, the scenarios considered in this study are described, focusing on the emergence and persistence of temporal quantum correlations in the three-state ion channel model. The subsequent section presents the results of numerical simulations, together with a detailed characterization and a possible interpretation consistent with the dynamics of the selective filter. Finally, the paper concludes with several directions for future investigation (*Results–Discussion–Conclusions*).

## Methods

### Model description

**Fig. 1:**
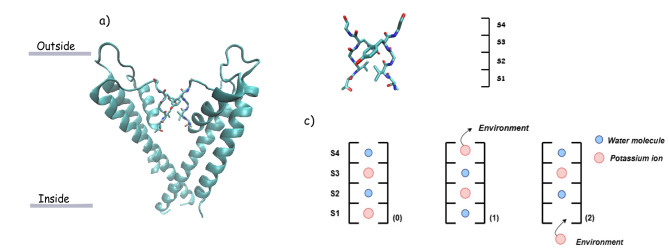
(**a**) Representation of KcsA ion channel with two of the four subunits (*PDB 1K4C*) obtained in Jmol; (**b**) A zoom representation of the selectivity filter with symbolic four K$^+$ ion-binding sites (S1–S4) with respective amino-acid order; (**c**) Schematic illustration of the ion-channel state configuration: *(0)= |0〉* (left), *(1)= |1〉* (middle), *(2)= |2〉* (right).

The phenomenological quantum model proposed in Ref^23^. and applied in this work is constructed for a channel, specifically the selective filter region, consisting of four sites^39^. Within this framework, three configurations of the ”knock-on^1^’ permeation mechanism is analyzed, wherein ions and water molecules transit the selectivity filter in a coordinated manner. A three-state system (see Fig. 1and compare with Fig.2 in Ref^23^.) is described as follows: (i) potassium ions occupy the first and third sites (S1–S3), while water molecules reside in the second and fourth sites (S2–S4); (ii) potassium ions are located in the second and fourth sites, with water molecules in the first and third sites; and (iii) in the transition from the previous state, a potassium ion is released to the environment, leaving space for another ion to enter the channel. The transition between these three configurations is associated with an energy barrier of electrostatic Coulomb-like origin^41^. The quantum model of the system is formulated^23^ in a three-dimensional Hilbert space with a basis denoted by *|i〉 , i=0,1,2* where *i* corresponds to the configurations described above.

The dynamics of the system (proposed in Ref^23^.) are governed by the time-independent Hamiltonian (setting *ℏ =1*):

1 $$\begin{aligned} H(t) = H_0 + c V, \quad H_0 = S_z, \end{aligned}$$

where *c* is the coherent hopping amplitude and *V* describes cyclic transitions between the three states. To capture the influence of the environment on ion transport, we explicitly model random perturbations of the hopping amplitudes that mimic fast, memoryless fluctuations of the surrounding medium. These fluctuations are represented by a classical white-noise process *ξ (t)* with zero mean and delta-function correlations. The effect of such noise is to introduce a stochastic modulation of the hopping constant *c*, which in turn leads to an additional fluctuating contribution to the Hamiltonian. Formally, this can be expressed as:

2 $$\begin{aligned} c \rightarrow c(t) = c \bigl (1 + \alpha \, \xi (t)\bigr ), \quad H_\textrm{noise}(t) = \alpha c \, \xi (t) V, \quad \mathbb {E}[\xi (t)] = 0, \quad \mathbb {E}[\xi (t)\xi (s)] = 2 \delta (t-s), \end{aligned}$$

where *α* controls the strength of the noise relative to the bare hopping rate *c*, *V* denotes the operator associated with the hopping process and $\mathbb {E}[\cdot ]$ is the expectation value on the realizations of the stochastic process.

Averaging over the stochastic realizations of the hopping fluctuations leads to a deterministic, ensemble-averaged master equation^42–44^,

3 $$\begin{aligned} \frac{d}{dt}\bar{\rho }(t) = -i [H_0 + c V, \bar{\rho }(t)] - c_1^2 [V, [V, \bar{\rho }(t)]]. \end{aligned}$$

Here, $\bar{\rho }(t)$ denotes the density matrix averaged over all realizations of the stochastic fluctuations, $H_0$ represents the intrinsic Hamiltonian of the system, and *V* is the operator associated with the hopping between sites in the channel. The parameter *c* sets the nominal hopping amplitude, while $c_1 = \alpha c$ quantifies the strength of decoherence induced by environmental noise.

Equation (3) can also be written in the Lindblad form, which is commonly used to describe open quantum systems:

4 $$\begin{aligned} \frac{d}{dt}\bar{\rho }(t) = -i [H_0 + c V, \bar{\rho }(t)] + \frac{c_1^2}{2} \Big ( - V^2 \bar{\rho } - \bar{\rho } V^2 + 2 V \bar{\rho } V \Big ). \end{aligned}$$

The first term, $-i [H_0 + c V, \bar{\rho }(t)]$, describes the coherent unitary evolution of the system, while the second term accounts for pure dephasing caused by rapid, memoryless fluctuations of the environment. This deterministic master equation preserves the physical consistency of the density matrix (positivity, Hermiticity, and normalization).

In the simulations, we explicitly distinguish these two contributions: *c* controls the coherent hopping dynamics, whereas $c_1$ can be adjusted independently to modulate the decoherence strength. This separation allows a systematic exploration of how environmental noise influences the persistence of quantum coherence and the potential violation of Leggett–Garg inequalities in the model system.

### Legget-Garg inequalities

One of the simplest Leggett–Garg inequalities (LGIs)^33^ can be expressed as:

5 $$\begin{aligned} K_3= & C_{21}+C_{32}-C_{31}\le 1 \end{aligned}$$

The quantity $K_3\equiv K_3(\tau )$ is composed by three-time correlation functions given by an expected value of an anti-commutator^28,33^ $C_{ji} =\frac{1}{2}\langle \{Q(t j ), Q(t i )\}\rangle$ of a dichotomous observable *Q*(*t*) calculated in the Heisenberg picture with corresponding measurement values *q(t) = ± 1* at three successive time instants $t_1< t_2 < t_3$. Here we consider *K* for a fixed inter-measurement time interval $\tau =t_2-t_1=t_3-t_2$ and set $t_1=0$.

For both closed and open quantum systems strictly Markovian time evolution of states *ρ (t)* can be written in terms of quantum dynamical semigroup^45^ $\rho (t)=\Lambda (t-t_0)\rho (t_0)$. Markovian approximation not only considerably simplifies a problem by neglecting system-environment entanglement^46,47^ but also in such a case the correlation functions in (5) for *t>s* can be calculated using^35,36^

6 $$\begin{aligned} C_{ij}= & \sum _{l,m=\pm 1}q_mq_l \text{ Tr }\{\Pi _m\Lambda (t_j-t_i)[\Pi _l\Lambda (t_i)\rho (0)\Pi _l] \} \end{aligned}$$

where $\{\Pi _m\}, m=\pm 1$ represent projective operators describing measurements of dichotomous observable *Q=Q(0)*. In an absence of dissipation the dynamical map $\Lambda =\exp (-iHs)$ is generated by a system Hamiltonian *H* whereas under more complicated and noisy conditions *Λ* is a solution of a suitable designed Master (Lindblad) equation^46,47^. This formulation allows one to interpret a potential violation of the inequality as a signature of non-classical temporal correlations in the system, highlighting deviations from classical.

### Simulation scenarios

To systematically explore the emergence and persistence of temporal quantum correlations in the modeled ion channel system, we compute the Leggett–Garg correlation function $K_3(\tau )$ under a variety of physically motivated conditions using the QuTiP package for quantum simulations^48,49^. These scenarios are designed to probe the sensitivity of quantum coherence to changes in system parameters, preparation procedures, and environmental influences. Specifically, we consider the following:

- **Different measurement operators.** We consider projective measurements onto the basis states, $P_i = \left| i\right\rangle \left\langle i\right|$ for *i = 0,1,2*, corresponding to the distinct channel configurations. The system is always initialized in the equal superposition state $\left| \psi _0\right\rangle = \frac{1}{\sqrt{3}}(\left| 0\right\rangle + \left| 1\right\rangle + \left| 2\right\rangle ),$ ensuring a consistent starting point for all measurements.
- **Variation of inter-state coupling strength.** The hopping strength parameter *c*, which sets the coherent transition rate (and thus the effective barrier height) between conformational states in the Hamiltonian, is varied (e.g., *c = 0.1, 0.3, 0.5*). Modifying *c* enables us to explore how different coupling strengths influence the violations of the Leggett–Garg inequalities.
- **Different initial states with fixed measurement.** Several initial states $\left| \psi _0^{(i)}\right\rangle$, specifically $\left| \psi _0^{(1)}\right\rangle , \left| \psi _0^{(2)}\right\rangle , \left| \psi _0^{(3)}\right\rangle$, are considered, while the measurement operator is fixed as the collective superposition $P=\sum _{i,j=0}^2 \left| i\right\rangle \!\left\langle j\right|$. For comparison, single-basis projectors are defined as $P_i=\left| i\right\rangle \!\left\langle i\right|$ for *i=0,1,2*.
- **Matched initial states and measurements.** For each initial state $\left| \psi _0^{(i)}\right\rangle$, the measurement operator is chosen as the corresponding projector $\Pi _i=\left| \psi _0^{(i)}\right\rangle \!\left\langle \psi _0^{(i)}\right|$. This matched configuration tests the system’s ability to maintain coherence under idealized preparation–measurement alignment.
- **Randomized hopping amplitude.** The initial state $\left| \psi _0\right\rangle = \frac{1}{\sqrt{3}}(\left| 0\right\rangle + \left| 1\right\rangle + \left| 2\right\rangle )$ is fixed, while the coherent hopping amplitude *c*, which sets the energy barrier between configurations in the system Hamiltonian, is drawn from a uniform distribution on the interval [0, 1]. For each value of *c*, the temporal correlation function $K_3(\tau )$ is computed using projectors $P_0, P_1,$ and $P_2$, and the results are averaged over 100 realizations. This procedure allows us to describe a*typical* pathway among a family characterized by different hopping and to capture the *typical* effect on temporal quantum correlations encoded on $K_3(\tau )$.
- **Noisy (open system) dynamics.** To account for environmental effects, we solve the ensemble-averaged master equation including a pure dephasing term $- c_1^2 [V, [V, \rho ]]$, where $V = \left| 1\right\rangle \left\langle 0\right| + \left| 2\right\rangle \left\langle 1\right| + \left| 0\right\rangle \left\langle 2\right|$. In modeling ion channels, we used one of two competing approaches, the so-called ”soft-knock” approach. This assumption implies that at most two selectively transported ions are present in the channel at any given time, and the main physical mechanism considered in the model is static ion repulsion. This approach describes the dynamics using *PT*-symmetric but not necessarily Hermitian time-evolution generators, analogous to those discussed in^23,50,51^. This was taken into account in the simulations, whose results include parameter regimes guaranteeing positivity of the dynamics. Verification involved checking the trace and positivity of the density matrix eigenvalues. The decoherence strength $c_1$ is varied (e.g., $c_1 = 0.0, 0.2, 0.3, 0.5$) to simulate an increase in environmental coupling, and the resulting dynamics are used to study how decoherence impacts the temporal correlations quantified by $K_3(\tau )$.

## Results

To explore how temporal quantum correlations manifest and persist in the three-state quantum model of the ion channel, we evaluate the Leggett–Garg correlation function $K_3(\tau )$ for the range of configurations introduced in the previous subsection (*Simulation Scenarios*). By systematically varying the measurement operators, the coherent coupling strength, the initial state preparation, and the level of environmental decoherence, we can disentangle the roles of coherent dynamics, measurement-induced disturbance, and noise in shaping the behaviour of $K_3(\tau )$. The following sections present the results for each scenario, highlighting regimes in which the Leggett–Garg inequality is violated and identifying the conditions that allow temporal quantum correlations to survive the longest.

### Different measurement operators

We examine temporal quantum correlations by evaluating the Leggett–Garg function $K_3(\tau )$ for three distinct projective measurements $P_i = \left| i\right\rangle \left\langle i\right|$ (*i = 0, 1, 2*), corresponding to channel configurations: (0) – potassium ions at sites S1 and S3, (1) – ions at sites S2 and S4 and (2) – the state released by ion at S1.

The initial state of the system, denoted $\left| \psi _0\right\rangle$, is fixed throughout the simulations. We consider a superposition $\left| \psi _0\right\rangle = \frac{1}{\sqrt{3}} (\left| 0\right\rangle + \left| 1\right\rangle + \left| 2\right\rangle )$, representing an equal probability amplitude for all three configurations. The Hamiltonian parameter *c* is fixed at *0.1*, setting the coherent transition rate between the states. This setup enables us to separate the influence of the initial state preparation from that of the projective measurement choice on the temporal correlations quantified by $K_3$.

**Fig. 2:**
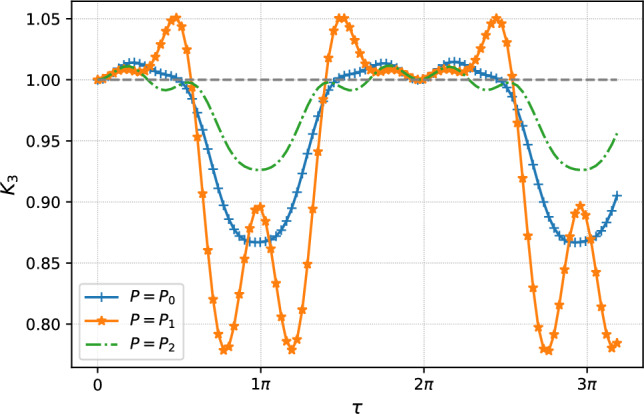
Time dependence of the Leggett–Garg correlation function $K_3(\tau )$ computed for the three projective measurements corresponding to configurations $P_0$ (blue), $P_1$ (orange), and $P_2$ (green) in the three-state quantum model of the ion channel; $\left| \psi _0\right\rangle = \frac{1}{\sqrt{3}} (\left| 0\right\rangle + \left| 1\right\rangle + \left| 2\right\rangle )$; the Hamiltonian parameter *c* is fixed at 0.1. The gray horizontal line denotes the classical bound $K_3 = 1$.

The results are depicted in Fig. 2. The strongest violations of the Leggett–Garg inequality occur for measurement $P_1$ (orange line), associated with potassium ions occupying the second and fourth sites (S2–S4), indicating pronounced temporal quantum correlations during this intermediate stage of the knock-on permeation process. These violations are regular and reach values as high as $K_3 \approx 1.06$, stronger than those observed for $P_0$ and $P_2$, suggesting that the configuration probed by $P_1$ exhibits more robust quantum effects, potentially reflecting an enhanced role of coherence.

In contrast, the measurement $P_2$ (green dashed line) barely crosses the classical boundary, with smaller oscillations and $K_3$ values remaining close to 1. This indicates that the configuration associated with $P_2$, which may follow ion emission and corresponds to the state with a potassium ion released from the channel, behaves more classically. The measurement $P_0$ (blue line) also shows LGI violations, but these are shallower and less frequent than for $P_1$. Overall, the configurations associated with $P_0$ and $P_2$ correspond to stages of the knock-on cycle where quantum signatures are less pronounced, likely reflecting initial or final phases of the process.

### Impact of inter-state hopping rate (variation of the parameter c)

**Fig. 3:**
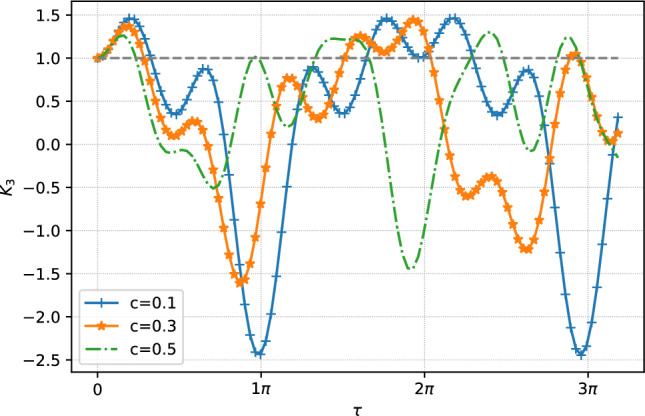
Time dependence of the Leggett–Garg correlation function $K_3(\tau )$ computed for various values of the hopping rate parameter: *c = 0.1* (blue), *c = 0.3* (orange), and *c = 0.5* (green); $\left| \psi _0\right\rangle = \frac{1}{\sqrt{3}} (\left| 0\right\rangle + \left| 1\right\rangle + \left| 2\right\rangle )$. The gray horizontal line indicates the classical bound $K_3 = 1$.

To investigate how the energy barrier between configurations affects temporal quantum correlations, we simulate the Leggett–Garg function $K_3(\tau )$ for various values of the coupling parameter *c*, which controls the transition amplitude between conduction states. The system is initialized in the state $\left| \psi _0\right\rangle = \frac{1}{\sqrt{3}}(\left| 0\right\rangle + \left| 1\right\rangle + \left| 2\right\rangle )$ and measured using a projector corresponding to a uniform superposition of all three configurations. Because both the initial state and the measurement operator span the entire configuration space, the amplitudes of different pathways interfere maximally, enhancing quantum effects. As a result, the oscillations of $K_3(\tau )$ are significantly larger, both above and below the classical bound of 1, than those observed for measurements on individual basis states. This can be clearly seen in Fig. 3, which demonstrates the enhanced oscillation amplitudes in comparison with the corresponding results for single-basis measurements shown in Fig. 2.

For each value of *c*, the function $K_3$ exhibits strong oscillations, with the largest violations above the classical bound $K_3 = 1$ reaching up to approximately 1.5 observed for *c = 0.1* (blue line). As *τ* increases, the dynamics of $K_3$ become increasingly differentiated between the various coupling strengths, with the case *c = 0.5* showing a particularly distinct behavior. Compared to the cases *c = 0.1* and *c = 0.3*, the $K_3$ dynamics for *c = 0.5* are more damped around $\tau = 2\pi$, suggesting that the system remains localized in a specific configuration for longer periods, thereby reducing temporal quantum correlations. Nevertheless, $K_3$ still exceeds the classical bound for *c = 0.5* over time. Overall, as the coupling parameter *c* increases, quantum signatures between configurations are gradually suppressed, leading to smaller oscillation amplitudes of $K_3(\tau )$, which, however, remain present over time.

### Different initial states with fixed measurement

**Fig. 4:**
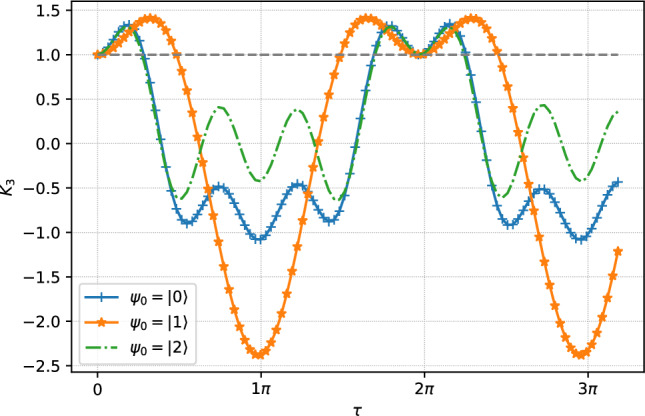
Violation of the Leggett–Garg inequality for different initial states in the three-state model of a four-site ion channel. $K_3(\tau )$ is plotted for different initial states: $\left| 0\right\rangle$ (S1–S3, blue), $\left| 1\right\rangle$ (S2–S4, orange), and $\left| 2\right\rangle$ (ion released, green) while the measurement operator is fixed as the collective superposition $P=\sum _{i,j=0}^2 \left| i\right\rangle \!\left\langle j\right|$. The gray horizontal line indicates the classical bound $K_3 = 1$.

To investigate how the system’s preparation affects quantum temporal correlations, we examine the Leggett–Garg inequality for different initial states. The results are presented in Fig. 4. The strongest violations of the Leggett–Garg inequality are observed for the initial state $\psi _0 = \left| 1\right\rangle$ (orange line), where the correlation function $K_3(\tau )$ exceeds the classical bound of 1 across several intervals of the evolution time *τ*, reaching values as high as $K_3 \approx 1.5$. These results indicate the manifestation of quantum effects in the dynamics when the system is prepared in this configuration.

Interestingly, the initial states $\left| 0\right\rangle$ (blue line) and $\left| 2\right\rangle$ (green dashed line) produce nearly identical patterns of LGI violations, although the amplitudes and durations for which $K_3>1$ are smaller than those observed for $\left| 1\right\rangle$. This symmetry likely arises from their analogous roles at the boundaries of the knock-on process, one preceding and the other following the central transition. The intermediate configuration $\left| 1\right\rangle$, on the contrary, exhibits significantly stronger violations of the Leggett–Garg inequality, consistent with its central position in the coherent transport cycle, where quantum coherence effects are maximally pronounced. The pronounced downward trend in $K_3(\tau )$ for the initial state $\left| 1\right\rangle$ highlights the enhanced sensitivity of temporal correlations during the intermediate knock-on configuration (S2–S4). This configuration represents the central stage of the ion transport process, where non-classical temporal correlations are strongest. The sharp decreases in $K_3(\tau )$, even when the values remain below the classical bound of 1, probably reflect transient decoherence or rapid changes in the underlying temporal correlations. In contrast, the initial states $\left| 0\right\rangle$ and $\left| 2\right\rangle$ correspond to the boundary configurations of the knock-on cycle. Their dynamics exhibit comparatively moderate temporal variations, producing smaller downward excursions of $K_3(\tau )$, although pronounced oscillations and fluctuations remain clearly visible.

### Matched initial states and measurements

**Fig. 5:**
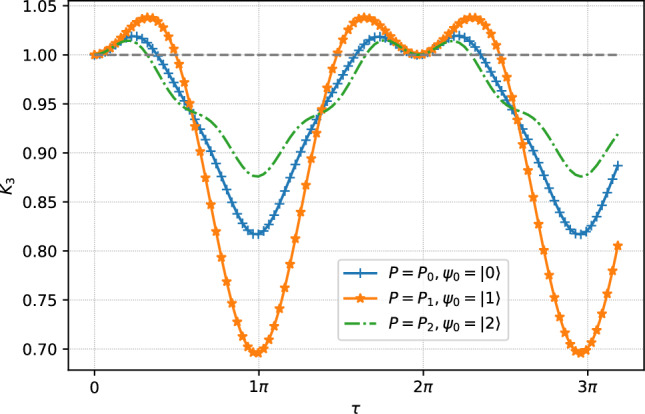
Violation of the Leggett–Garg inequality for different initial states in the three-state model of a four-site ion channel, with the projective measurement operator *P* matched to the initial state $\psi _0$. Curves correspond to $P = P_0,\, \psi _0 = \left| 0\right\rangle$ (blue), $P = P_1,\, \psi _0 = \left| 1\right\rangle$ (orange), and $P = P_2,\, \psi _0 = \left| 2\right\rangle$ (green). The gray horizontal line indicates the classical bound $K_3 = 1$.

The aim of this analysis is to investigate how temporal quantum correlations depend on the interplay between system preparation and measurement alignment. For each configuration $\left| \psi _0^{(i)}\right\rangle$, the projective measurement operator is chosen as $\Pi ^{(i)} = \left| \psi _0^{(i)}\right\rangle \left\langle \psi _0^{(i)}\right|$, ensuring maximal overlap with the prepared state. This matched setting allows us to probe the extent to which nonclassical temporal correlations can be preserved and revealed when the measurement is optimally tuned to the initial state. By comparing the Leggett–Garg correlation function $K_3(\tau )$ across the three configurations of the knock-on cycle, our aim is to identify the dynamic stages most sensitive to *measurement-induced disturbances* and the decay of temporal correlations due to decoherence under idealized preparation–measurement conditions.

The results obtained for the matched initial states and measurement operators are shown in Fig. 5. Violations of the Leggett–Garg inequality occur at the same evolution times *τ* approximately for the three configurations, indicating that the temporal structure of coherent dynamics is preserved throughout the knock-on cycle. However, the magnitude of these violations varies significantly: the configuration $P_1$ and $\psi _0 = \left| 1\right\rangle$ exhibits the strongest departures from the classical bound. The $P_0$ configuration also shows LGI violations, though the amplitudes are smaller, while $P_2$ yields the weakest deviations from the classical bound, with smallest drops below unity. This hierarchy suggests that the intermediate configuration $P_1$ with $\psi _0 = \left| 1\right\rangle$ again exhibits the strongest temporal quantum correlations, $P_0$ with $\psi _0 = \left| 0\right\rangle$ shows an intermediate level and $P_2$ with $\psi _0 = \left| 2\right\rangle$ shows the weakest manifestation of quantum effects relevant to LGI violation within the coherent transport cycle.

### Randomized hopping amplitude.

**Fig. 6:**
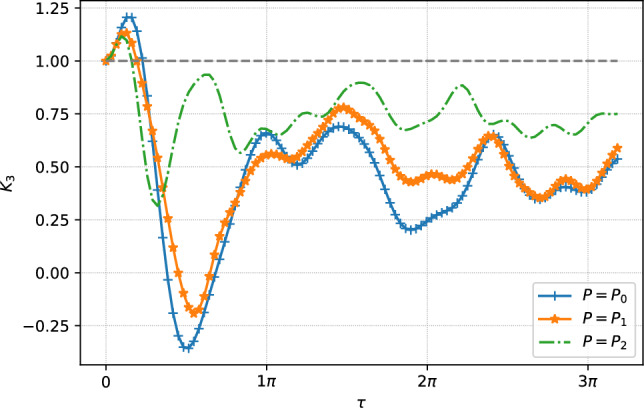
Leggett–Garg correlator $K_3(\tau )$ for different measurement operators $P_0$, $P_1$, and $P_2$ in the three-state ion channel model with dynamic decoherence. The hopping rate *c* is randomly drawn from a normal distribution, then a unitary trajectory is calculated for each realization. The final results are averaged over 100 realizations. The gray horizontal line indicates the classical bound $K_3 = 1$.

Figure 6 illustrates the Leggett–Garg correlation function $K_3(\tau )$ for projective measurements $P_0$ (blue), $P_1$ (orange) and $P_2$ (green dashed) in the presence of averaging with respect to the parameter c of the system Hamiltonian. Although violations of the classical bound $K_3 \le 1$ are still observed, they are significantly reduced in both magnitude and duration compared to the fluctuation-free case. The largest exceedances occur for $P_0$ and $P_1$. Averaging also leads to smoother oscillations and faster decay of correlation over time. This behaviour reflects the decohering impact of certain values of *c* in the ensemble of channels characterized by their system Hamiltonians, which reduce but not removes the observed quantum signatures encoded in the violation of the LGI. It is worth emphasizing that here the ”decohering noise” is modeled as static fluctuations of the parameter: the hopping rate *c* is randomly drawn from a uniform distribution, then a unitary trajectory (without time-dependent decoherence) is calculated for each realization, and finally the results are averaged over all realizations. This procedure is analogous to ensemble averaging in a sample: each channel has a different fixed parameter, but within a given realization the evolution remains quantum. Randomization of *c* (as implemented in the code) captures: (i) the natural heterogeneity of the channels, and (ii) fast variations of the parameters controlling the hopping. Averaging over these realizations produces an effective decoherence: interference peaks are smoothed, and LGI violations occur less frequently and with smaller amplitudes. The differences between $P_0$, $P_1$, and $P_2$ remain: some configurations (here $P_0$ and $P_1$) still provide favorable conditions for coherence at certain values of *c*, resulting in larger average violations, while $P_2$ exhibits the smallest effect in overcoming the classical bound and stands out most clearly from the other cases.

### Noisy (open system) dynamics

**Fig. 7:**
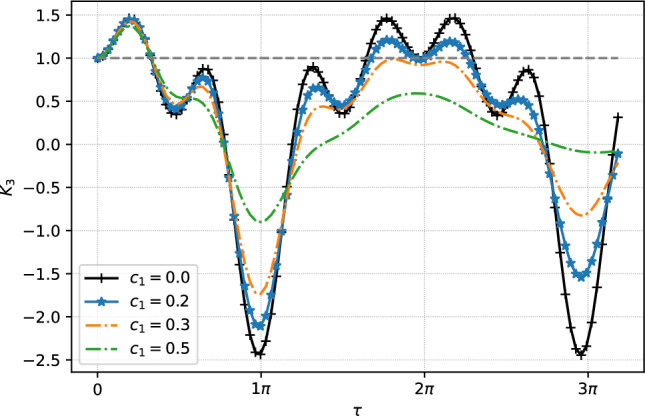
Violation of the Leggett–Garg inequality in the three-state ion channel model. The decoherence strength $c_1$ is varied through the Lindblad term. The curves correspond to $c_1 = 0$ (black), $c_1 = 0.2$ (blue), $c_1 = 0.3$ (orange), and $c_1 = 0.5$ (green dashed); $\left| \psi _0\right\rangle = \frac{1}{\sqrt{3}} (\left| 0\right\rangle + \left| 1\right\rangle + \left| 2\right\rangle )$, and the measurement operator is fixed as the collective superposition $P=\sum _{i,j=0}^2 \left| i\right\rangle \!\left\langle j\right|$. The gray horizontal line indicates the classical bound $K_3 = 1$.

In contrast to the static disorder case, where fluctuations in the hopping rate lead to averaging over different coherent trajectories, the inclusion of dynamic decoherence via a Lindblad term continuously suppresses quantum superpositions during the evolution. In this scenario (see Fig. 7), the hopping parameter *c* in the Hamiltonian is kept fixed, while the strength of the decoherence $c_1$ is varied independently through a Lindblad term. This separation allows us to observe how environmental noise alone affects the quantum dynamics without altering the coherent transport rate.

As $c_1$ increases, the $K_3(\tau )$ curves are increasingly suppressed, reflecting the continuous loss of quantum properties during the evolution. The state with the highest decoherence, $c_1 = 0.5$, shows the fastest decay and loses the ability to violate the Leggett–Garg inequality much sooner than states with other (lower) $c_1$ values. In contrast, the state with minimal decoherence, $c_1 = 0.0$, retains oscillatory behavior for a longer duration, indicating that coherent dynamics dominate when environmental noise is absent.

The differences in the shapes of the curves provide insights into the decoherence mechanism: higher $c_1$ not only reduces the amplitude of oscillations but also modifies their phase and frequency slightly, demonstrating how dynamic decoherence acts on the system independently of the hopping process. These observations highlight that dynamic decoherence, controlled via $c_1$, directly impacts the temporal quantum correlations captured by $K_3(\tau )$. As $c_1$ increases, not only are the oscillation amplitudes damped, but the intervals during which the Leggett–Garg inequality is violated become shorter. This illustrates that environmental noise alone can limit the persistence of non-classical correlations, independently of the coherent hopping dynamics set by *c*.

## Discussion

In this work, we investigate the violation of the Leggett–Garg inequality within a three-state ion channel model, highlighting the factors that govern temporal quantum correlation. The model was originally introduced by Seifi et al.^23^, who analyzed a three-state ion channel system within the Lindblad framework to explore the relation between hopping rates and the persistence of quantum coherence. Building upon this foundation, our approach employs Leggett–Garg inequality simulations to probe the genuinely quantum nature of the system’s dynamics–specifically, to identify the conditions under which the system’s quantumness is maximized. To this end, we systematically explore the presence and robustness of violation of the simplest Legett-Garg inequality under different operational regimes. We consider variations in measurement bases and initial states, as well as stochastic modifications of the hopping amplitude that determine the energy barriers between configurations in the selectivity filter. Environmental effects are incorporated through a controllable dissipation rate within the Lindblad master equation, allowing us to examine how open-system dynamics affect non-classical temporal correlations.

For reference, the system occupies three configurations: *|0 〉* (K$^+$ at S1–S3, water at S2–S4), *|1 〉* (K$^+$ at S2–S4, water at S1–S3), and *|2 〉*, representing ion release and channel vacancy for a new ion. Among the studied cases, the configuration corresponding to the intermediate state $\left| 1\right\rangle$–where potassium ions occupy the central sites (S2–S4)–emerges as particularly important for the knock-on mechanism. Across most measurement scenarios ($P_1$–$P_3$) and for initial states $\psi _0 = \left| 1\right\rangle , \left| 2\right\rangle , \left| 3\right\rangle$ or their superpositions (see Fig. 2, 4, 5), it is associated with the most pronounced violations of the classical bound $K_3 \le 1$. Serving as the crucial transition between ion entry and exit, this intermediate configuration forms a balanced state that exhibits pronounced nonclassical features. In the state *|1 〉*, the system occupies an intermediate configuration in which potassium ions are located at sites S2 and S4, flanked by water molecules at S1 and S3. This arrangement allows multiple transport pathways whose probability amplitudes can interfere constructively and destructively. As a result, correlations may exhibit non-classical features depending on the measurement and system parameters. In our simulations, the projective measurement $P_1$ yields the most pronounced violations of the Leggett–Garg inequality, displaying the strongest depressions of $K_3(\tau )$ below the classical bound, and thus emerges as the dominant contributor to the observed non-classical behavior. In contrast, the measurement $P_2$, associated with the configuration $\left| 2\right\rangle$, exhibits significantly weaker violations, with only small exceedances of $K_3$ above the ’classical limit’ equal to 1.

In the original work by Seifi et al^23^., the state $\left| 1\right\rangle$ was identified as a short-lived transient configuration. In the absence of environmental noise (*γ =0*), the population of $\left| 1\right\rangle$ undergoes oscillations but decays to nearly zero over time, reflecting its role as an intermediary state. The energy less favorable state $\left| 1\right\rangle$ is quickly depopulated, resulting in long-term dynamics primarily governed by the entry states $\left| 0\right\rangle$ and exit - $\left| 2\right\rangle$ states.

Our analysis based on Leggett–Garg inequality simulations highlights a complementary aspect of $\left| 1\right\rangle$. Although short-lived in terms of population dynamics, this configuration provides the most favorable conditions for quantum signatures: the symmetric arrangement of ions and water molecules enables constructive and destructive overlap of transport pathways. As a result, the population $P_1$ exhibits the strongest violations of the LG inequality, with $K_3(\tau )$ dropping also significantly below the classical bound. By comparison, the $\left| 2\right\rangle$configuration, associated with ion release and partial channel dehydration, displays only weak violations. This interpretation aligns with Seifi et al^23^., who reported that after the decoherence time the system predominantly relaxes into $\left| 2\right\rangle$, the lowest-energy state, reflecting the channel’s natural tendency to cross and release ions to the environment. Thus, while $\left| 1\right\rangle$ emerges as the primary carrier of quantum coherence, $\left| 2\right\rangle$ seems to represent the more classical-like and energetically favorable endpoint of the transport cycle.

In real transport phenomena, the interplay between potassium ions and water molecules within the channel can be related to *dewetting* phenomena and hydrophobic gating mentioned as important mechanism in several papers^52–54^. The authors postulate that dehydration of the selective filter cavity is a key step in the gating procedure of potassium channels using different activation mechanisms. Hydrophobic gating is a mechanism in which a narrow, hydrophobic region within an ion channel can switch between a conductive (water-filled) and a non-conductive (dewetted) state. Dewetting occurs when water is expelled from this region due to unfavorable interactions with hydrophobic surfaces, creating an energetic barrier that inhibits ion permeation. The presence of water molecules stabilizes the conductive state by lowering the free-energy barrier for ion translocation, and their absence promotes gating closure. This process is influenced by the channel geometry, local electrostatics, and the presence of permeant ions. Regarding considered three-state model, in the case of state *|1 〉*, where potassium ions are symmetrically positioned and water molecules occupy alternating sites, the channel remains well hydrated. This hydration supports ion mobility, facilitates coherent transport, and enables interference between multiple transport pathways–associated with strong Leggett–Garg inequality violations. In state *|2 〉*, partial ion depletion increases the likelihood of dewetting in the selectivity filter, leading to reduced water occupancy and an additional energetic barrier to ion motion. This suppresses the hopping rate and accelerates decoherence, producing weaker non-classical correlations. State *|0 〉* represents the initial phase of ion entry, with hydration and coherence levels intermediate between *|1 〉* and *|2 〉*.

Our results also indicate that measurements in superposition bases lead to stronger violations of the Leggett–Garg inequality compared to purely projective measurements, even when the initial states coincide with the corresponding projectors (compare Figs. 4 and 5). In the model, this reflects the fact that the system is not rigidly projected onto a single ion configuration in the selectivity filter, but instead is described by overlapping possibilities of site occupancy. In this situation, probability amplitudes associated with different transport pathways can interfere constructively or destructively, thereby enhancing coherence effects and producing larger deviations of $K_3$ beyond the classical bound. By contrast, projective measurements enforce a collapse to one of the basis states, which reduces interference and diminishes non-classical signatures.

In the considered framework of ion channel dynamics, both the preparation of the initial state and the choice of measurement protocol can influence the degree of quantum correlations. In particular, the tendency of superposition measurements to yield stronger LGI violations indicates that dynamical superpositions of ionic configurations may enhance nonclassical features in transport and extend their temporal persistence. However, this interpretation should be treated with caution, as the strongest violations arise from the model measurement formalism and do not necessarily imply that similar effects would occur in the physical system, where many intermediate states are present.

The dependence of $K_3(\tau )$ on the hopping amplitude *c* highlights how energy barriers between ion configurations shape temporal quantum correlations. From the perspective of ion-channel dynamics, this suggests that the coupling strength between binding sites plays a non-trivial role. As emphasized by Seifi *et al.*, a sufficiently high hopping rate is a prerequisite for effective selective transport. Importantly, despite the presence of decoherence, a large hopping amplitude sustains the system in a coherent regime. In our analysis, the amplitude by which the $K_3$ function exceeds the classical bound decreases with increasing *c*, while the frequency of bound violations over time becomes sustained. This may indicate that strong hopping does not enhance the instantaneous strength of temporal quantum correlations but instead contributes to their persistence, suggesting a more robust–though less pronounced–form of nonclassical behavior (see Fig. 3). A qualitatively similar tendency is observed in the presence of stochastic fluctuations of the hopping rate (Fig. 6), where LGI violations persist but are confined to the initial stages of the dynamics. This further underlines that fluctuations do not eliminate nonclassical features, but rather restricts them to short time windows, in line with the general trend that stronger perturbations reduce the amplitude and duration of temporal quantum correlations.

While static fluctuations of the hopping rate *c* correspond to averaging over coherent trajectories with fixed parameters, dynamic decoherence introduced via a Lindblad term represents a continuous suppression of quantum superpositions during the evolution. In our case, the hopping rate is fixed (*c=0.1*), and decoherence is independently controlled by the parameter $c_1$, which allows us to isolate the impact of environmental noise (see Fig. 7). As $c_1$ increases, $K_3(\tau )$ is progressively damped, with higher values leading to both faster decay and earlier loss of LGI violations. This behavior reflects the characteristic role of an open quantum system: while coherent dynamics can persist in the absence of noise, coupling to an external environment steadily erodes temporal quantum correlations. Thus, whereas strong hopping is often associated with maintaining coherence in transport scenarios^23^, our analysis highlights that in the LGI framework, the balance between coherent transport and environmental decoherence must be considered explicitly, as noise alone can suppress nonclassical signatures regardless of the hopping amplitude.

The primary objective of this study was to investigate the behavior of a theoretical three-state model in the quantum regime and assess its capacity to exhibit temporal quantum correlations trough the violations of the Leggett–Garg inequality. While the model successfully captures these fundamental features, its correspondence with the full biophysical complexity of potassium channels remains to be more thoroughly explored. Nevertheless, the framework holds great potential, as the study of LGI violations highlights which aspects of ionic transport–such as coherent hopping, intermediate configurations, and interference between pathways–may prove most promising and insightful for future investigations. A special attention in this matter can be devoted to dewetting phenomena, which, together with quantum properties, are of considerable interest in the context of ion transfer through the selectivity filter. A short-lived ”dewetted” state of the selectivity filter can transiently impede ion conduction by introducing an energetic barrier, thereby modulating the kinetics of ion permeation and affecting both selectivity and gating mechanisms. Investigating these events experimentally and theoretically offers a promising avenue for understanding how water-mediated structural fluctuations influence ion transport, and may help identify the conditions under which coherence or interference effects become most pronounced. Integrating such insights with detailed molecular dynamics or multi-site quantum models could therefore provide a more complete picture of the interplay between hydration dynamics and quantum transport phenomena in realistic ion channels. In light of these findings, future studies integrating more detailed approaches, such as molecular dynamics simulations or extended quantum models, could further elucidate how microscopic transport mechanisms and environmental fluctuations influence nonclassical features in realistic channels, thereby bridging the gap between abstract theoretical predictions and the complex biophysical reality of ion conduction.

## Conclusions

Analysis of the three-state model of ion channel permeation reveals violations of the Leggett–Garg inequalities, providing insight into how specific ion configurations and hopping dynamics influence temporal quantum correlations. In particular, the intermediate state $\left| 1\right\rangle$ acts as a transient but crucial mediator of quantum effects between transport pathways, whereas the states $\left| 0\right\rangle$ and $\left| 2\right\rangle$ dominate in regimes characterized by reduced nonclassical features.

Within the model, both the choice of measurement basis and the presence of environmental noise are found to strongly influence the observed LGI violations. Superposition-based measurements enhance nonclassical temporal correlations by enabling interactions between overlapping configurations, whereas projective measurements tend to suppress such effects. The static fluctuations of the hopping parameters restrict LGI violations to shorter time intervals, while dynamic decoherence progressively diminishes the violations regardless of the hopping amplitude.

These results provide a conceptual perspective and suggest directions for investigating biologically relevant processes, such as ion–water interactions, hydration dynamics, and dewetting events. LGI analyses improve the identification of stages with the most pronounced temporal quantum correlations, offering insight into the potential role of quantum effects in ion transport. The investigation of temporal correlations highlights regions of the system’s energetic landscape that are most relevant for quantum behavior, with the short-lived intermediate configuration in the knock-on cycle emerging as a particularly promising target for future studies of quantum effects in ion conduction.

## Data Availability

The datasets and source codes used and/or analysed during the current study are available from the corresponding author on reasonable request.

## Footnotes

1 In the direct knock-on mechanism, K$^+$ ions permeate through the selectivity filter without water, forming direct ion–ion contacts within the conserved filter structure. In contrast, the soft knock-on mechanism assumes co-permeation of water molecules, which separate the K$^+$ ions as they move through the filter^40^.
